# Long-Term Administration of Dienogest for the Treatment of Pain and Intestinal Symptoms in Patients with Rectosigmoid Endometriosis

**DOI:** 10.3390/jcm9010154

**Published:** 2020-01-06

**Authors:** Fabio Barra, Carolina Scala, Umberto Leone Roberti Maggiore, Simone Ferrero

**Affiliations:** 1 Academic Unit of Obstetrics and Gynecology, IRCCS Ospedale Policlinico San Martino, Largo R. Benzi 10, 16132 Genoa, Italy; fabio.barra@icloud.com; 2 Department of Neurosciences, Rehabilitation, Ophthalmology, Genetics, Maternal and Child Health (DiNOGMI), University of Genoa, 16132 Genoa, Italy; 3 Unit of Obstetrics and Gynecology, Gaslini Institute, 16147 Genova, Italy; carolinascala@icloud.com; 4 Department of Gynecologic Oncology, IRCCS National Cancer Institute, 20133 Milan, Italy; ulrm@libero.it; 5 Piazza della Vittoria 14 S.r.l., 16121 Genova, Italy

**Keywords:** rectosigmoid endometriosis, dienogest, hormonal treatment, pain, intestinal symptoms

## Abstract

Background: Rectosigmoid endometriosis is a severe form of deep endometriosis, which may be responsible for pain symptoms and a wide range of intestinal complains such as diarrhea, constipation, and abdominal cramping. The primary objective of this study was to evaluate the efficacy of long-term therapy with dienogest (DNG), a fourth-generation progestin, for ameliorating quality of life, pain, and intestinal symptoms of patients affected by rectosigmoid endometriosis. Methods: A retrospective analysis of a prospectively collected database was done on patients with endometriotic nodules infiltrating at least the muscular layer of the rectosigmoid wall with an estimated colorectal stenosis <60%. The diagnosis of rectosigmoid endometriosis was based on transvaginal ultrasonography. Patients accepting to participate in the study received continuous oral treatment with DNG at the dose of 2 mg/day. Results: At the end of the first year of treatment, a significant decrease in dysmenorrhea (*P* < 0.001), chronic pelvic pain (*P* < 0.001), dyspareunia (*P* < 0.001), dyschezia (*P* < 0.001), and in intestinal symptoms (*P* < 0.001) was observed. A progressive increase of the Endometriosis Health Profile-30 (EHP-30) and Gastrointestinal Quality of Life Index (GIQLI) scores was observed in the first two years of therapy (*P* < 0.001 and *P* < 0.001, respectively). Improvements of patients’ symptoms and quality of life were maintained until the end of the study. The regimen was well tolerated, and the frequency and amount of irregular bleeding decreased as treatment progressed. Conclusion: The current study confirms the efficacy of long-term therapy with DNG for treating symptoms caused by rectosigmoid endometriosis.

## 1. Introduction

Rectosigmoid endometriosis is defined by the presence of deep endometriotic nodules infiltrating at least the muscularis propria of the rectosigmoid wall; it may affect between 4% and 37% of women with endometriosis [1,2]. Despite being sometimes asymptomatic, rectosigmoid endometriosis can cause pain and a wide range of intestinal symptoms such as diarrhea, constipation, abdominal bloating, passage of mucus in the stools, and rectal bleeding [1]; these complaints may sometimes mimic irritable bowel syndrome, leading to a significant delay in diagnosis [3]. Transvaginal ultrasonography can accurately diagnose the presence of rectosigmoid endometriosis and estimate the depth of infiltration of the nodules in the intestinal wall [4]. For this reason, it should be considered the first-line investigation in patients with suspicion of deep endometriosis [5]. Surgical management of rectosigmoid endometriosis (shaving excision, discoid resection, or segmental bowel resection) leads to a significant improvement of pain and intestinal complains [6,7]. Overall, these procedures may carry a not-negligible risk of complications such as ureteral damages, damages to the pelvic nerves, and dehiscence of the suture in cases of bowel resection. Women affected by smaller intestinal nodules without evidence of significant stenosis of the bowel lumen or subocclusive symptoms can be treated by medical therapies. These treatments can improve patient’s pain and intestinal complains, thus avoiding or postponing surgery [8,9,10].

The current first-line options for medical treatment of endometriosis include combined oral contraceptives and single progestins [11]. The latter drug class has been growingly adopted in this setting [12]. Dienogest (DNG) is an oral fourth-generation progestin with anti-ovulatory activity and also a direct antiproliferative and anti-inflammatory effects on ectopic endometriotic cells, by modulation of prostaglandin production and metabolism [13]. In previous studies, this drug was demonstrated to be effective in relieving endometriosis-related symptoms, including the intensity of dysmenorrhea, dyspareunia, and chronic pelvic pain [14]. However, no study specifically evaluated the efficacy of long-term administration DNG for conservatively treating patients with rectosigmoid endometriosis. The primary objective of this study was to evaluate the efficacy of 36-month therapy with DNG for ameliorating quality of life, pain, and intestinal symptoms of patients with rectosigmoid endometriosis. Secondary objectives were the assessment of the effect of this therapy in modifying the size of endometriotic nodules and treatment tolerability.

## 2. Methods

### 2.1. Study Design

This study was based on the retrospective analysis of a prospectively collected database. Women of reproductive age who had a diagnosis of rectosigmoid endometriosis were treated at our Institution between January 2014 and December 2018. Patients were informed that there is limited evidence on the effects of hormonal treatments on the symptoms due to bowel endometriosis [8,9,10,15]. They were informed that, in some patients, the volume of the intestinal nodule may increase during treatment and that medical therapy for endometriosis should not be considered definitively curative [16,17]. An informed written consent for the use of clinical data for scientific purposes was obtained from the patients.

### 2.2. Study Population

This study included women with pain and intestinal symptoms of more than 6 months duration. Demographic data (such as age, parity, previous hormonal and surgical therapies for endometriosis) were collected. The diagnosis of rectosigmoid endometriosis was based on transvaginal ultrasonography (TVS). Only women with endometriotic nodules infiltrating at least the muscular layer of the rectal wall and with an estimated stenosis of the bowel lumen <60% were considered eligible to be treated by hormonal therapy, as previously suggested [18]. Although some of the patients included in the study had previously undergone laparoscopy for endometriosis, none of them had previous bowel surgery (with the exception of appendectomy). Contraindications for hormonal treatment were desire to conceive, obstructive uropathy, or evidence of complex adnexal cysts, contraindications for receiving estrogens and progestogen, use of drugs that interfere with contraceptive steroid metabolism, abnormal findings at breast examination and mammary imaging. Exclusion criteria for the study were medical regimens for endometriosis other than nonsteroidal anti-inflammatory drugs in the three months before administration of DNG (six months for gonadotropin-releasing hormones analogues (GnRH-a)), psychotic disorders, history of drug or alcohol abuse.

### 2.3. Study Protocol

Patients received continuous oral treatment with DNG (Visanne^®^; Bayer, Milan, Italy) at the dose of 2 mg/day.

### 2.4. Assessment of Symptoms

Before starting the treatment and after 6, 12, 24, and 36 months of treatment, the presence and severity of pain symptoms (dysmenorrhea, chronic pelvic pain, and deep dyspareunia) were evaluated by using a 10 cm visual analogue scale (VAS). At baseline and at follow-up consultations, women completed a symptom analogue scale questionnaire (1 indicated an absence of symptoms; 10 indicated the highest score) evaluating the following intestinal symptoms: dyschezia, diarrhea, constipation, intestinal cramping, and passage of mucus. At baseline and at the follow-up consultations, the GIQLI (Gastrointestinal Quality of Life Index) and EHP-30 (Endometriosis Health Profile-30) questionnaires were used to assess patients’ gastrointestinal-specific health-related and the overall quality of life, respectively. The GIQLI is a 36-item gastrointestinal-specific questionnaire with five subscales (GI symptoms, emotion, physical function, social function, and medical treatment) as well as a total score. Higher scores represent better health-related quality of life and subscores range from 0 to 4 while the total score ranges from 0 to 144 [19]. The EHP-30 questionnaire comprises two parts. A core questionnaire that is based on five scales (pain, control and powerlessness, emotional well-being, social support, and self-image), globally containing a total of 30 items. This section is applicable to all women with endometriosis. A modular questionnaire, which does not necessarily apply to all women with endometriosis, consists of six additional scales (work, relationship with children, sexual intercourse, infertility, medical profession, and treatment) and contains a total of 23 items. Items within scales are summed to create a raw score, and then each scale is translated into a score ranging from 0 (best health status) to 100 (worst health status). This scaled score is equal to the total of the raw scores of each item in the scale divided by the maximum possible raw score of all the items in the scale, multiplied by 100 [20].

Adverse effects were investigated at each consultation. At the time of prescribing DNG, patients were invited to report adverse effects by telephone call or by email and they were informed of the possibility of undergoing a consultation in case the adverse effect was judged to be significant.

### 2.5. Ultrasonographic Assessment of Rectosigmoid Endometriotic Nodules

TVS was performed by an experienced ultrasonographer (over 2000 exams in patients with deep endometriosis) by using a Voluson E6 machine (GE Healthcare Ultrasound, Milwaukee, WI, USA). The presence of rectosigmoid endometriosis was confirmed by rectal water-contrast transvaginal ultrasonography (RWC-TVS) [21]. Briefly, a flexible catheter was introduced through the anus into the rectal lumen up to 15 cm from the anus. Up to 350 mL of sterile saline solution was injected into the rectosigmoid by using a syringe connected to the catheter.

Transvaginal ultrasonographic was performed at each follow-up consultation. Rectovaginal endometriosis infiltrating the rectum was defined as a solid, regular, or irregular, hypoechogenic mass that was adherent and/or penetrated into the intestinal wall distorting and/or replacing the normal appearance of the muscularis propria, hypoechoic or hyperechoic foci were sometimes present [1]. The presence of other deep endometriotic nodules and endometriomas was assessed at baseline and each follow-up visit.

The largest diameter and the volume of the rectosigmoid nodule were evaluated at each ultrasonographic assessment. The volume of the nodules was estimated by using virtual organ computer-aided analysis (VOCAL, GE Healthcare). The VOCAL technique was used to obtain a sequence of 20 sections of each endometriotic nodule around a fixed axis, each after 9° rotation from the previous section. The contour of each nodule was drawn manually by using the roller ball cursor of the ultrasound machine.

### 2.6. Statistical Analysis

The volumetric changes of the rectosigmoid endometriotic nodules were evaluated by using the Student’s *t*-test and Mann–Whitney U test according to the data distribution. The normal distribution of continuous variable data was calculated with the Kolmogorov–Smirnov test. Categorical data were analyzed by using the χ^2^ test or the Fisher’s exact test. *P* < 0.05 was considered statistically significant. Data were analyzed by using SPSS software version 24.0 (SPSS Science, Chicago, IL, USA).

### 2.7. Ethical Approval

The study protocol was approved by the Hospital Research Review Committee (236REG2019; approval 1/2019).

## 3. Results

Eighty-three patients were included in the study and started the treatment with DNG. Table 1 reports the demographic characteristics of the patients. At baseline, 88.0% (73/83) of patients had dysmenorrhea, 72.3% (60/83) had chronic pelvic pain, 86.4% (57/66) had dyspareunia; 69.9% (58/83) had at least one intestinal symptom, among which the most commonly experienced was dyschezia (82.8%, 48/58). The intestinal symptoms of the study population are presented in Supplementary Table S1. Overall, 30 (36.1%) and 55 patients (66.3%) had undergone previous surgery or hormonal therapy for endometriosis. There was no significant difference in baseline symptoms, general, and gastrointestinal quality of life between patients who underwent or did not undergo previous surgery or hormonal therapy for endometriosis (Supplementary Table S2).

**Figure 1:**
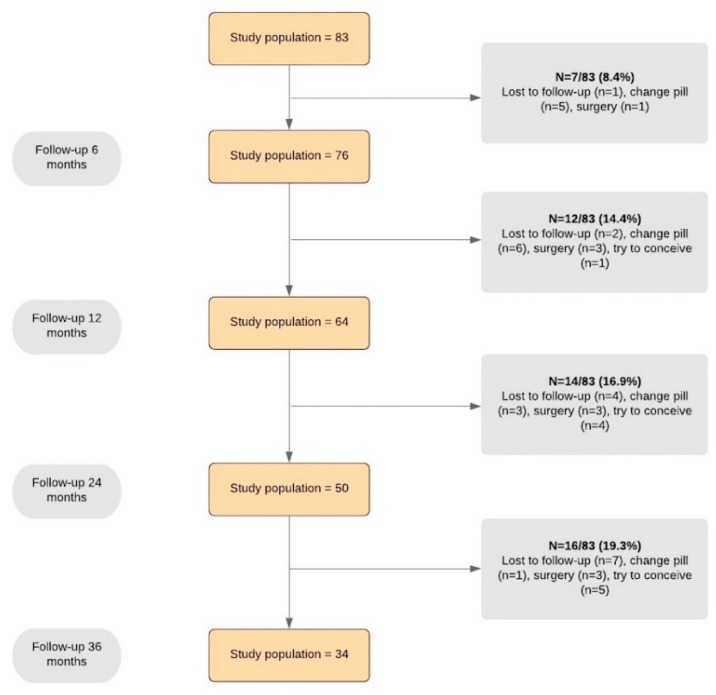
Flow chart showing women’s progress through the study.

The diagrammatic flow of the participants is given in Figure 1. 

At the 36-month follow-up, 34 women (41.0%) were still using DNG. With the exclusion of patients lost to follow-up, discontinuation because of adverse events was the most common cause of withdrawing (37.1%, 13/35). Discontinuation because of adverse effects was more frequent in the first year of therapy (47.3%, 9/19) in comparison to the second year (14.3%, 2/14, *P =* 0.046) and third year of therapy (12.5%. 2/16; *P =* 0.035). After treatment discontinuation, the patients most commonly opted to change the hormonal regimen (15/35; 9/15 because of adverse events; 4/15 for unchanged or worsened disease symptoms; 2/15 because of desire of contraception) or to undergo surgery (10/35; 6/10 because of adverse events; 4/10 because of unchanged or worsened disease symptoms).

### 3.1. Pain and Gastrointestinal Symptoms 

Table 2 shows the changes in pain and intestinal symptoms scores during the therapy with DNG. After 6 months of treatment, there was a significant improvement in the severity of dysmenorrhea (*P <* 0.001), chronic pelvic pain (*P <* 0.001), deep dyspareunia (*P <* 0.001) in comparison with baseline. In particular, dysmenorrhea was completely abolished in patients achieving a status of amenorrhea (63.6%, 49/77) induced by the progestin.

After 12 months, a further amelioration of these symptoms was observed (*P =* 0.046, *P =* 0.001, and *P =* 0.045, respectively, in comparison with 6-month values). These symptoms remained stable without statistically significant differences at 24 (*P =* 0.632 and *P =* 0.582 and *P =* 0.909) and 36 (*P =* 0.732 and *P =* 0.819 and *P =* 0.384) months of treatment.

After 6 months of treatment, there was a significant amelioration in the intensity of most intestinal symptoms, such as dyschezia (*P <* 0.001), constipation (*P <* 0.001), diarrhea (*P <* 0.001), feeling of incomplete evacuation (*P <* 0.001), and intestinal cramping (*P <* 0.001 compared with baseline). After 12 months of treatment, a significant improvement of passage of mucus (*P <* 0.001) and a further improvement of dyschezia (*P =* 0.039) in comparison of the previous follow-up consultation were observed. The intensity of intestinal symptoms remained stable without statistically significant differences with previous values until 36 months of treatment. At 6 months of the therapy, there was also the almost complete disappearance of intestinal symptoms related to the menstrual cycle, such as constipation during the menstrual cycle (*P <* 0.001), diarrhea during the menstrual cycle (*P =* 0.009) and cyclical rectal bleeding (*P =* 0.047).

### 3.2. Patients’ Quality of Life

After 6 months of treatment, there was a statistically significant difference in the global EHP-30 score (from 80.7 ± 7.1 to 74.4 ± 8.5 points; *P <* 0.001), despite a not-significant increase of self-image (*P =* 0.876) and control and powerlessness (*P =* 0.077) core subdomains in comparison to baseline. After 12 months of treatment, EHP-30 score was further ameliorated in all subdomains in comparison to 6-month follow-up (global score 57.0 ± 12.1; *P <* 0.001), and it remained stable at 24 and 36 months of treatment (*P =* 0.253 and *P =* 0.768, respectively). 

A statically increase of GIQLI score was observed from baseline to 12 months (from 90.0 ± 7.2 to 102.0 ± 8.5; *P <* 0.001). A further improvement was observed at 24 months (113.3 ± 5.7; *P <* 0.001 in comparison to previous value). GIQLI remained stable during the last year of study treatment (*P =* 0.079). 

**Figure 2:**
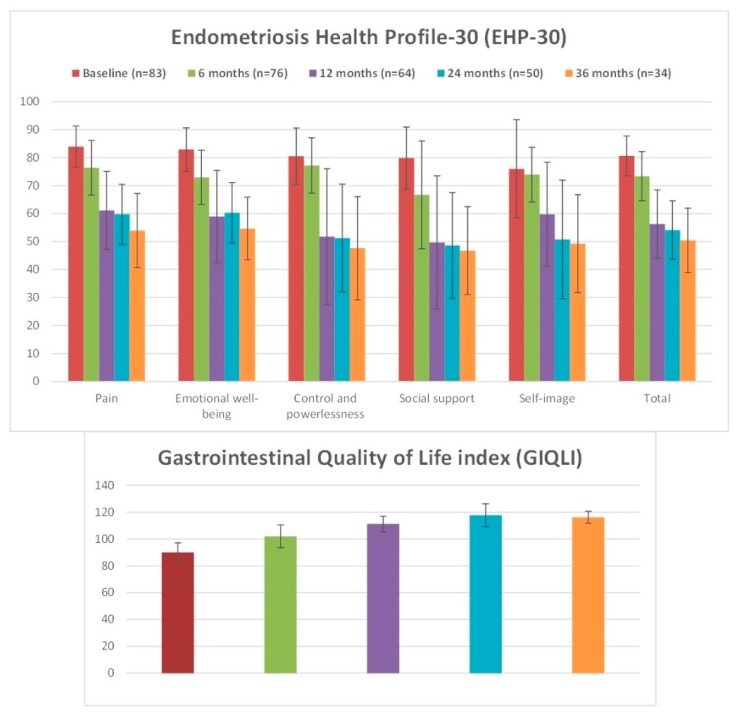
Representation of changes in Endometriosis Health Profile-30 (EHP-30) and Gastrointestinal Quality of Life Index (GIQLI) scores during the treatment.

Figure 2 and Supplementary Table S3 report the changes in patients’ general and gastrointestinal quality of life scores during the therapy with DNG.

### 3.3. Size of Rectosigmoid Endometriotic Nodules 

Table 3 shows the changes in the size of endometriotic nodules during the therapy with DNG. After 6 months of treatment, the volume of the endometriotic nodules significantly decreased when compared with baseline values (from 4.0 ± 2.1 to 3.7 ± 1.4 cm^3^, *P =* 0.033). After the end of the first year of treatment, a statistical decrease of the largest diameter observed (from 2.7 ± 0.5 cm at baseline to 2.4 ± 0.5 cm, *P =* 0.046) and a further significant reduction in mean nodule volume was observed (3.1 ± 1.2 cm^3^; *P <* 0.001 when compared with baseline and *P <* 0.001 when compared with 6 month); moreover, the mean ± SD reduction of largest diameter and volume in comparison to baseline was 1.1 ± 1.7 cm and 16.8 ± 36.7 cm^3^, respectively.

After 24 and 36 months of treatment, the largest diameter and volume of endometriotic nodules remained stable (*P =* 0.421, *P =* 0.856 and *P =* 0.153, *P =* 0.560, respectively). 

At the end of the study, in 52.9% (18/34) of patients a decrease of volume of at least 10%, in 35.3% (12/34) of them an unchanged volume (±10%), and in 11.8% (4/34) of them an increase was observed in comparison to baseline values.

### 3.4. Tolerability of the Treatment

Overall, 52 adverse events were recorded in 47 patients (56.6%). Table 4 reports the adverse events during the study. The most common of them were weight gain (30.1%; 21/52) and abnormal uterine bleeding (26.9%; 14/52). In particular, the prevalence of abnormal uterine bleeding was 16.9% (13/77) after 6 months of treatment and decreased thereafter to only 10.8% (10/64) after 12 months, 6% (3/50) after 24 months, and 2.9% (1/34) after 36 months. Abnormal uterine bleeding (46.2%; 6/13) and weight gain (30.8%; 4/13) were the most common adverse events responsible for treatment interruption; the other adverse events responsible for discontinuation are reported in Supplementary Table S4.

## 4. Discussion

Several previous studies investigated the use of hormonal drugs, such as low-dose oral contraceptive pill [15], norethisterone acetate (NETA) [8,9], desogestrel, triptorelin [10], and letrozole [8] for treating patients with bowel endometriosis, demonstrating that these hormonal therapies are effective in ameliorating pain, intestinal symptoms, and global quality of life of these women.

In the current study, we observed that DNG therapy led to significant decrease in dysmenorrhea, chronic pelvic pain, dyspareunia, and dyschezia in the first 12 months of therapy, which was maintained for the other two years until the end of the study (Table 2); similar findings were observed for intestinal symptoms, with a progressive increase in the global GIQLI score in the first 24 months of therapy (Figure 2). To the best of our knowledge, this is the first study evaluating the long-term administration of DNG in a large population of women with rectosigmoid endometriosis. 

Surgery is obviously required in women with rectosigmoid endometriosis at high risk of occlusion, such as patients with ultrasonographic bowel stenosis >60% or suffering subocclusive symptoms, and those with doubtful diagnosis with suspicion of bowel malignancy [22]. Furthermore, hormonal treatments for endometriosis are contraceptive or do not allow conception and, therefore, bowel surgery may be required in patients wishing to conceive who do not tolerate pain and intestinal symptoms. 

The responsiveness of deep intestinal lesions to progestins is supported by the presence of progesterone receptors in ectopic glands infiltrating the muscular layer of the bowel wall [23]. DNG binds to the progesterone receptor with high specificity and produces a potent progestogenic effect related to the high circulating levels of the unbound molecule [24]. In addition, this drug has potent antiproliferative activity on the endometrium, as well as anti-inflammatory and anti-angiogenic properties [25]. A systematic review including nine randomized trials on the use of DNG versus placebo or GnRH-analogues demonstrated that, regardless of the dose, DNG was an effective drug for controlling pain in women with deep endometriosis with no major side effects, such as bone mass reduction, as happens with GnRH analogues, a fact that tends to contraindicate their use in long-term regimens [14]. Moreover, in some studies, this drug was significantly better tolerated [26] and efficacious [27] than NETA for treating patients with deep endometriosis.

Two previous studies assessed the effect of DNG in patients with bowel endometriosis. In prospective cohort trial, Leonardo-Pinto et al. evaluated its efficacy for 12 months in 30 women affected with rectovaginal and bowel endometriosis. At baseline, participants reported persistent pain complaints despite medical treatment with other progestins; intestinal pain significantly decreased during the study period without a significant reduction in bowel lesion size. However, this study is limited by the fact that the authors did not specify whether all the patients had intestinal lesions as well as the specific bowel locations of the lesions [28]. In another trial by Vercellini et al., the use of NETA or DNG was employed for treating patients with colorectal endometriosis (*n* = 50) in comparison to surgical treatment (*n* = 37), obtaining both similar symptom improvement and satisfaction. However, in this trial, a specific analysis for patients receiving DNG was not done [29]. 

It has been previously reported that hormonal therapies can decrease the size of deep endometriotic nodules [18,30]; however, it seems that there is no direct correlation between improvement of symptoms and decrease in nodule volume [30]. In our study, at the end of follow-up visits under DNG therapy, a mean nodule volume reduction of at least 10% was reported by around 30% of women. Otherwise, 5–10% of patients had an increase in the volume of endometriotic nodules, nevertheless often not presenting a worsening of clinical symptoms. This observation is not surprising; in clinical practice, it is common to observe extensive intestinal lesions in patients who have been treated for years by hormonal therapies (particularly combined oral contraceptives) [16]. The non-response of some lesions may be due to the structural feature of the deep nodules that may contain extensive fibrosis [31]. On the basis of this evidence, patients affected by bowel endometriosis undergoing hormonal therapies should be informed of the potential risk of progression of the disease during long-term medical management [16]. Overall, medical therapy for deep endometriosis should not be considered curative and patients should start the pharmacological regimen with the understanding that may still require a surgical treatment in future [29]. Current evidence suggests the need for a routine ultrasonographic monitoring of patients undergoing hormonal treatment. Imaging exams should not be performed only when patients report a worsening of clinical symptoms because deep endometriotic nodules may grow despite the improvement in symptoms. 

In our study, one of most common adverse effects related to DNG treatment was irregular uterine bleeding, consistent with previous reports [28,32]. As expected, its frequency and amount decreased as treatment progressed (from 16.9% of patients at 6 months to 6% at 36 months). In general, treatment-related adverse effects were moderate and the majority of the study population tolerated them. Moreover, in line with previous studies [32,33], the rate of patients experiencing amenorrhea was around 65%, and this status correlated to the almost complete disappearance of menstrual-related symptoms, and, in particular, dysmenorrhea. It is important to underscore that progestins, including DNG, are frequently associated with bloating, mood change, weight gain, and irregular bleeding [23], which may adversely impact on long-term treatment adherence. This important aspect must be clarified during counseling, together with the fact that conservative surgery as an isolated measure does not guarantee definitive symptom relief. Moreover, when side effects are poorly tolerated, a switch to a different drug may also be discussed. As the higher percentage of exit from the study was observed within the first year of treatment, this period may be particularly important for selecting those women who will accept long-term DNG therapy.

This study had some strengths: firstly, the sample was the one of the largest in studies investigating the administration of DNG for deep endometriosis; secondly, the duration of DNG use was longer than in previous studies with a follow-up under treatment until 36 months. Otherwise, we are also aware that some limitations characterize this study: firstly, its design is retrospective, although data have been prospectively collected in a standardized fashion for clinical follow-up; moreover, the diagnosis of rectosigmoid nodules was based on RWC-TVS and not on laparoscopy and subsequent histology. Therefore, the concomitant involvement of right colon, cecum, and small bowel in some studied women cannot be excluded. These results cannot be generalized to all women; in fact, the women enrolled in this study were highly motivated to start a medical therapy due to the severe symptomatology associated with rectosigmoid endometriosis so that they decided avoiding alternative treatments, such as surgery. This high motivation could also explain the low withdrawal rate despite the not-negligible percentage of side effects recorded during the treatment.

## 5. Conclusions

The current study confirmed the efficacy of long-term DNG therapy in treating symptoms caused by rectosigmoid endometriosis. In particular, this regimen allows to improve pain and intestinal complaints as well as an amelioration of global patients’ quality of life with good tolerability. Moreover, it can cause a slight reduction in the size of endometriotic nodules. DNG as a long-term regimen was demonstrated to have an acceptable safety-profile.

## Figures and Tables

**Table 1:** Demographic characteristics of the patients included in the study.

	Patients (*n* = 83)
Age (years; mean ± SD)	32.8 ± 5.0
BMI (kg/m^2^; mean ± SD)	21.0 + 3.0
Smokers (*n*, %)	15 (18.1%)
Parity (*n*, %)	23 (27.7%)
Sexually active (*n*, %)	66 (81.9%)
Other imaging methods confirming the diagnosis of rectosigmoid endometriosis	63 (80.7%)
*Magnetic resonance imaging*	56 (83.6%)
*Computerized tomography colonography*	8 (11.9%)
*Multidetector computerized tomography enema*	2 (3.1%)
*Double-contrast barium enema*	1 (1.5%)
Previous surgery for endometriosis (*n*, %)	30 (36.1%)
*Ablation of endometriotic implants*	18 (60%)
*Excision of endometriotic nodules*	12 (43.3%)
*Removal of endometriotic ovarian cysts*	6 (23.3%)
Time to previous surgery for endometriosis (months; median, interval)	43 (9–87)
Previous hormonal therapy for endometriosis (*n*, %)	55 (66.3%)
*Estroprogestins*	22 (73.3%)
*Other progestins*	11 (36.7%)
*Danazol*	4 (13.3%)
*GnRH agonists*	2 (6.7%)
Time to previous hormonal therapy for endometriosis (months; median, interval)	6.7 (4–37)

GnRH—gonadotrophin-releasing hormone analogues; BMI—body mass index.

**Table 2:** Changes in pain and gastrointestinal symptoms during the treatment.

Symptom	Baseline	6 Months	12 Months	24 Months	36 Months
Dysmenorrhea	7.0 ± 1.2 (*n* = 73)	5.5 ± 1.4 (*n* = 27) *P <* 0.001 *	4.3 ± 1.8 (*n* = 18) *P <* 0.001 * *P =* 0.046 ^§^	4.3± 2.1 (*n* = 13) *P <* 0.001 * *P =* 0.044 ^§^ *P =* 0.632 ^¥^	4.4 ± 2.0 (*n* = 8) *P <* 0.001 * *P =* 0.146 ^§^ *P =* 0.546 ^¥^ *P =* 0.732 ^£^
Chronic pelvic pain	6.1 ± 0.9 (*n* = 60)	4.0 ± 1.3 (*n* = 53); *P <* 0.001 *	3.3 ± 1.0 (*n* = 44); *P <* 0.001 * *P =* 0.001 ^§^	3.4 ± 1.6 (*n* = 35); *P <* 0.001 * *P =* 0.548 ^§^ *P =* 0.582 ^¥^	3.3 ± 1.2 (*n* = 22); *P <* 0.001 * *P =* 0.245 ^§^ *P =* 0.763 ^¥^ *P =* 0.819 ^£^
Deep dyspareunia	5.7 ± 1.9 (*n* = 57)	4.3 ± 2.2 (*n* = 53); *P <* 0.001 *	3.6 ± 1.8 (*n* = 45); *P <* 0.001 * *P =* 0.045 ^§^	3.6 ± 1.6 (*n* = 38); *P <* 0.001 * *P =* 0.538 ^§^ *P =* 0.909 ^¥^	3.7 ± 1.1 (*n* = 26); *P <* 0.001 * *P =* 0.563 ^§^ *P =* 0.226 ^¥^ *P =* 0.384 ^£^
Dyschezia	5.2 ± 1.6 (*n* = 48)	3.8 ± 2.2 (*n* = 43); *P <* 0.001 *	3.2 ± 2.6 (*n* = 40); *P <* 0.001 * *P =* 0.039 ^§^	3.0 ± 1.8 (*n* = 31); *P <* 0.001 * *P =* 0.908 ^§^ *P =* 0.131 ^¥^	2.7 ± 1.9 (*n* = 19); *P <* 0.001 * *P =* 0.304 ^§^ *P =* 0.220 ^¥^ *P =* 0.398 ^£^
Constipation	6.7 ± 1.5 (*n* = 37)	2.4 ± 2.4 (*n* = 33); *P <* 0.001 *	1.9 ± 2.0 (*n* = 33); *P <* 0.001 * *P =* 0.086 ^§^	1.9 ± 1.6 (*n* = 25); *P <* 0.001 * *P =* 0.942 ^§^ *P =* 0.192 ^¥^	1.8 ± 1.7 (*n* = 16); *P<*0.001 * *P =* 0.609 ^§^ *P =* 0.300 ^¥^ *P =* 0.436 ^£^
Constipation during the menstrual cycle	4.6 ± 1.5 (*n* = 19)	1.9 ± 0.6 (*n* = 8) *P <* 0.001 *	0.8 ± 0.3 (*n* = 6) *P =* 0.001 * *P =* 0.012 ^§^	1.4 ± 0.4 (*n* = 5) *P =* 0.015 * *P =* 0.012 ^§^ *P =* 0.059 ^¥^	1.1 ± 0.1 (*n* = 3) *P =* 0.063* *P =* 0.272 ^§^ *P =* 0.195 ^¥^ *P =* 0.423 ^£^
Diarrhea	6.9 ± 1.4 (*n* = 36)	2.2 ± 2.3 (*n* = 33) *P<*0.001 *	1.9 ± 2.1 (*n* = 33); *P <* 0.001 * *P =* 0.086 ^§^	1.8 ± 1.5 (*n* = 25); *P <* 0.001 * *P =* 0.878 ^§^ *P =* 0.129 ^¥^	1.6 ± 1.8 (*n* = 16); *P <* 0.001 * *P =* 0.359 ^§^ *P =* 0.108 ^¥^ *P =* 0.223 ^£^
Diarrhea during the menstrual cycle	4.0 ± 1.6 (*n* = 15)	1.6 ± 0.8 (*n* = 7) *P =* 0.009 *	1.2 ± 0.4 (*n* = 6) *P =* 0.006 * *P =* 0.434 ^§^	1.4 ± 1.1 (*n* = 5); *P =* 0.040 * *P =* 0.807 ^§^ *P =* 0.865 ^¥^	1.0 ± 0.0 (*n* = 3); *P =* 0.015 * *P =* 0.020 ^§^ *P =* 0.321 ^¥^ *P =* 0.423 ^£^
Intestinal cramping	5.6 ± 2.0 (*n* = 40)	3.5 ± 2.3 (*n* = 37); *P <* 0.001 *	3.2 ± 1.4 (*n* = 29); *P <* 0.001 * *P =* 0.079 ^§^	3.1 ± 1.6 (*n* = 24); *P <* 0.001 * *P =* 0.036 ^§^ *P =* 0.953 ^¥^	3.1 ± 1.5 (*n* = 17); *P <* 0.001 * *P =* 0.141 ^§^ *P =* 0.725 ^¥^ *P =* 0.681 ^£^
Feeling of incomplete evacuation	5.6 ± 1.7 (*n* = 44)	3.6 ± 2.4 (*n* = 43); *P <* 0.001 *	3.2 ± 2.0 (*n* = 36); *P <* 0.001 * *P =* 0.455 ^§^	3.3 ± 1.9 (*n* = 31); *P <* 0.001 * *P =* 0.588 ^§^ *P =* 0.792 ^¥^	3.2 ± 1.5 (*n* = 20); *P <* 0.001 * *P =* 0.128 ^§^ *P =* 0.609 ^¥^ *P =* 0.499 ^£^
Passage of mucus	5.6 ± 1.9 (*n* = 43)	5.5 ± 2.2 (*n* = 41); *P =* 0.206 *	2.9 ± 1.8 (*n* = 34); *P <* 0.001 * *P <* 0.001 ^§^	3.0 ± 2.3 (*n* = 29); *P <* 0.001 * *P =* 0.001 ^§^ *P =* 0.977 ^¥^	2.9 ± 1.1 (*n* = 19); *P<*0.001 * *P =* 0.001 ^§^ *P =* 0.861 ^¥^ *P =* 0.954 ^£^
Cyclical rectal bleeding	4.1 ± 2.3 (*n* = 11)	1.3 ± 0.9 (*n* = 6) *P =* 0.047 *	1.3 ± 0.5 (*n* = 4) *P =* 0.057 * *P =* 0.330 ^§^	0.8 ± 0.3 (*n* = 3); *P =* 0.004 * *P =* 0.188 ^§^ *P =* 0.177 ^¥^	1.2 ± 0.8 (*n* = 3); *P =* 0.053 * *P =* 0.873 ^§^ *P =* 0.635 ^¥^ *P =* 0.275 ^£^

* Compared with baseline; ^§^ compared with 6-month follow-up; ^¥^ compared with 12-month follow-up; ^£^ compared with 24-month follow-up. NA: complete absence. The number of patients suffering each symptom at baseline and at the specific follow-up visits is reported in parenthesis.

**Table 3:** Changes in main largest diameter and volume of rectosigmoid nodules during the treatment.

Largest Diameter of Endometriotic Nodules (mm ± SD)				
Baseline	6-month treatment	12-month treatment	24-month treatment	36-month treatment
2.7 ± 0.5	2.6 ± 0.5 *P =* 0.075 *	2.4 ± 0.5 *P =* 0.003 * *P =* 0.046 ^§^	2.3 ± 0.6 *P =* 0.026 * *P =* 0.073 ^§^ *P =* 0.421 ^¥^	2.3 ± 0.5 *P =* 0.038 * *P =* 0.234 ^§^ *P =* 0.421 ^¥^ *P =* 0.856 ^£^
**Volume of Endometriotic Nodules (cm^3^ ± SD)**				
Baseline	6-month treatment	12-month treatment	24-month treatment	36-month treatment
4.0 ± 2.1	3.7 ± 1.4 *P =* 0.033 *	3.1 ± 1.2 *P <* 0.001 * *P <* 0.001 ^§^	3.2 ± 1.3 *P =* 0.006 * *P =* 0.017 ^§^ *P =* 0.153 ^¥^	3.0 ± 1.5 *P =* 0.011 * *P =* 0.010 ^§^ *P =* 0.677 ^¥^ *P =* 0.560 ^£^

* Compared with baseline; ^§^ compared with 6-month follow-up; ^¥^ compared with 12-month follow-up; ^£^ compared with 24-month follow-up.

**Table 4:** Adverse events (any grade) experienced during the treatment.

Adverse Events (*n* = 52)	Number of Adverse Effects (%)	Prevalence of Adverse Effects in the Study Population (*n* = 83) (%)
Weight gain	21 (30.1)	25.3
Abnormal uterine bleeding *	14 (26.9)	16.9
Headache	11 (21.2)	13.3
Depression	5 (9.6)	6.0
Decrease libido	2 (3.8)	2.4
Acne	1 (1.9)	1.2

* Consisting in irregular bleeding cycle or intermenstrual spotting/bleeding.

## Supplementary Materials

The following are available online at https://www.mdpi.com/2077-0383/9/1/154/s1, Table S1: Gastrointestinal symptoms at baseline, Table S2: Baseline symptoms and quality of life scores in patients who did or did not undergo previous surgery and hormonal therapy for endometriosis, Table S3: Changes in patients’ general and gastrointestinal quality of life during the treatment, Table S4: Adverse events responsible for treatment interruption (*n* patients = 13).
