# Supplementary material for: Long-Term Administration of Dienogest for the Treatment of Pain and Intestinal Symptoms in Patients with Rectosigmoid Endometriosis
Source: J Clin Med. 2020 Jan 6;9(1):154. doi: 10.3390/jcm9010154 (PMC7019573; doi:10.3390/jcm9010154)
Supplement: Supplementary file 1 [file jcm-09-00154-s001.zip › Supplementary Table 3.docx]

**Supplementary Table 3.** Changes in patients’ general and gastrointestinal quality of life during the treatment

| **EHP-30** | | | | | |
| --- | --- | --- | --- | --- | --- |
| **Core domain** | Baseline  (n= 83) | 6-month  (n= 76) | 12-month  (n= 64) | 24-month  (n= 50) | 36-month  (n= 43) |
| **Pain** | 84.0 ±7.3 | 76.4 ± 9.8 *P<*0.001* | 61.1 ± 13.9 *P<*0.001*  *P<*0.001^§^ | 59.8 ± 10.7 *P<*0.001*  *P<*0.001^§^  *P=*0.977^¥^ | 54.0 ± 13.2 *P<*0.001*  *P<*0.001^§^  *P=*0.981^¥^  *P=*0.090^£^ |
| **Control and powerlessness** | 80.6 ±10.1 | 79.1 ± 9.2 *P=*0.077* | 53.9 ± 23.2 *P<*0.001*  *P<*0.001^§^ | 51.7 ± 18.5 *P<*0.001*  *P<*0.001^§^  *P=*0.419^¥^ | 49.0 ± 18.6 *P<*0.001*  *P<*0.001^§^  *P=*0.624^¥^  *P=*1.000^£^ |
| **Social support** | 79.9 ±11.0 | 66.7 ± 19.3 *P<*0.001* | 49.7 ± 23.8 *P<*0.001*  *P<*0.001^§^ | 48.6 ± 18.9 *P<*0.001*  *P<*0.001^§^  *P=*0.655^¥^ | 49.7 ± 15.1 *P<*0.001*  *P=*0.003^§^  *P=*0.421^¥^  *P=*0.681^£^ |
| **Emotional well-being** | 82.9 ±7.7 | 72.9 ± 9.7 *P<*0.001* | 59.0 ± 16.5 *P<*0.001*  *P<*0.001^§^ | 60.3 ± 10.8 *P<*0.001*  *P<*0.001^§^  *P=*0.687^¥^ | 54.6 ± 11.2 *P<*0.001*  *P<*0.001^§^  *P=*0.372^¥^  *P=*0.001^£^ |
| **Self-image** | 76.0 ±17.5 | 77.0 ± 10.2 *P=*0.876* | 61.2 ± 19.0 *P<*0.001*  *P<*0.001^§^ | 53.7 ± 20.5 *P<*0.001*  *P<*0.001^§^  *P=*0.040^¥^ | 54.7 ± 15.7  *P<*0.001*  *P<*0.001^§^  *P=*0.328^¥^  *P=*0.854^£^ |
| **Global score** | 80.7 ±7.1 | 74.4 ± 8.5 *P<*0.001* | 57.0 ± 12.1 *P<*0.001*  *P<*0.001^§^ | 54.8 ± 10.0 *P<*0.001*  *P<*0.001^§^  *P=*0.253^¥^ | 53.1 ± 11.3 *P<*0.001*  *P<*0.001^§^  *P=*0.768^¥^  *P=*0.361^£^ |
| **GIQLI** | | | | | |
| **Global score** | 90.0 ± 7.2 | 102 ± 8.5  *P<*0.001* | 111.3 ± 5.7 *P<*0.001*  *P<*0.001^§^ | 117.8 ± 8.5 *P<*0.001*  *P<*0.001^§^  *P<*0.001^¥^ | 116.2 ± 4.4 *P<*0.001*  *P<*0.001^§^  *P=*0.001^¥^  *P=*0.079^£^ |

* compared with baseline; ^§^ compared with 6-month follow-up ^¥^ compared with 12-month follow-up ^£^ compared with 24-month follow-up.
