# Supplementary material for: Long-Term Administration of Dienogest for the Treatment of Pain and Intestinal Symptoms in Patients with Rectosigmoid Endometriosis
Source: J Clin Med. 2020 Jan 6;9(1):154. doi: 10.3390/jcm9010154 (PMC7019573; doi:10.3390/jcm9010154)
Supplement: Supplementary file 1 [file jcm-09-00154-s001.zip › Supplementary Table 2.docx]

**Supplementary Table 2.** Baseline symptoms and quality of life scores in patients who underwent or did not undergo previous surgery and hormonal therapy for endometriosis

| **Symptom** | **Previous surgery** | **Not Previous surgery** | **P** | **Previous hormonal therapy** | **Not Previous hormonal therapy** | **P** |
| --- | --- | --- | --- | --- | --- | --- |
| Dysmenorrhea | 7.0 ± 1.2  (n=27) | 7.1 ± 1.2  (n=46) | 0.82 | 7.2 ± 1.0  (n=47) | 6.8 ± 1.4  (n=26) | 0.12 |
| Chronic pelvic pain | 6.0 ± 1.1  (n=24) | 6.1 ± 0.8  (n=36) | 0.53 | 6.1 ± 1.0  (n=38) | 6.0 ± 0.8  (n=22) | 0.59 |
| Deep dyspareunia | 5.8 ± 2.0  (n=20) | 5.6 ± 1.8  (n=37) | 0.74 | 5.5 ± 2.0  (n=37) | 6.1 ± 1.4  (n=20) | 0.28 |
| Dyschezia | 5.1 ± 1.4  (n=16) | 5.3 ± 1.7  (n=32) | 0.80 | 5.4 ± 1.5  (n=29) | 5.0 ± 1.8  (n=19) | 0.36 |
| Constipation | 6.6 ± 1.6  (n=8) | 6.5 ± 1.4  (n=29) | 0.59 | 6.4 ± 1.5  (n=8) | 6.4 ± 1.6  (n=29) | 0.88 |
| Constipation during the menstrual cycle | 4.2 ± 0.3  (n=4) | 4.3 ± 1.7  (n=15) | 0.52 | 4.4 ± 1.5  (n=13) | 4.5 ± 0.7  (n=6) | 0.31 |
| Diarrhoea | 6.8 ± 1.5  (n=8) | 7.1 ± 1.2  (n=28) | 0.62 | 6.8 ± 1.3  (n=24) | 7.0 ± 1.6  (n=12) | 0.71 |
| Diarrhoea during the menstrual cycle | 3.6 ± 1.3  (n=2) | 4.0 ± 1.7  (n=12) | 0.74 | 4.2 ± 1.7  (n=9) | 3.4 ± 1.4  (n=5) | 0.36 |
| Intestinal cramping | 5.5 ± 2.1  (n=16) | 5.6 ± 2.0  (n=24) | 0.89 | 5.4 ± 2.1  (n=25) | 5.9 ± 1.8  (n=15) | 0.39 |
| Feeling of incomplete evacuation | 5.3 ± 1.8  (n=18) | 5.8 ± 1.7  (n=26) | 0.40 | 5.4 ± 1.7  (n=28) | 5.9 ± 1.7  (n=16) | 0.41 |
| Passage of mucus | 5.7 ± 1.8  (n=16) | 5.7 ± 1.9  (n=27) | 0.90 | 5.5 ± 1.9  (n=26) | 5.9 ± 1.8  (n=17) | 0.41 |
| Cyclical rectal bleeding | 4.1 ± 2.9  (n=5) | 4.2 ± 2.0  (n=6) | 0.97 | 3.6 ± 2.1  (n=8) | 5.6 ± 2.7  (n=3) | 0.22 |
| Global GQLI | 90.3 ± 5.9  (n=30) | 89.9 ± 7.8  (n=53) | 0.84 | 89.1 ± 7.8  (n=55) | 92.0 ± 5.4  (n=28) | 0.08 |
| Global EHP-30 | 82.5 ± 8.0  (n=30) | 79.6 ± 6.4  (n=53) | 0.08 | 81.5 ± 7.3  (n=55) | 79.0 ± 6.5  (n=28) | 0.05 |
