# Supplementary material for: Long-Term Administration of Dienogest for the Treatment of Pain and Intestinal Symptoms in Patients with Rectosigmoid Endometriosis
Source: J Clin Med. 2020 Jan 6;9(1):154. doi: 10.3390/jcm9010154 (PMC7019573; doi:10.3390/jcm9010154)
Supplement: Supplementary file 1 [file jcm-09-00154-s001.zip › Supplementary Table 4.docx]

**Supplementary Table 4.** Adverse events responsible for treatment interruption (n patients=13)

| **Adverse Events** | Number (%) |
| --- | --- |
| Abnormal uterine bleeding | 6 (46.2) |
| Weight gain | 4 (30.8) |
| Headache | 2 (13.3) |
| Depression | 2 (13.3) |
| Acne | 1 (6.6) |
| **Total events** | 15 |
