# Supplementary material for: Long-Term Administration of Dienogest for the Treatment of Pain and Intestinal Symptoms in Patients with Rectosigmoid Endometriosis
Source: J Clin Med. 2020 Jan 6;9(1):154. doi: 10.3390/jcm9010154 (PMC7019573; doi:10.3390/jcm9010154)
Supplement: Supplementary file 1 [file jcm-09-00154-s001.zip › Supplementary Table 1.docx]

**Supplementary Table 1.** Gastrointestinal symptoms at baseline

|  | Patients  (n = 83) |
| --- | --- |
| **At least a gastrointestinal symptom** | 58 (69.9) |
| Dyschezia (n, %) | 48 (57.8) |
| Incomplete evacuation (n, %) | 44 (53.0) |
| Passage of mucus (n, %) | 43 (51.8) |
| Intestinal cramping (n, %) | 40 (48.2) |
| Constipation (n, %) | 37 (44.6) |
| Diarrhoea (n, %) | 36 (43.4) |
| Constipation during the menstrual cycle (n, %) | 19 (22.9) |
| Diarrhoea during the menstrual cycle (n, %) | 15 (18.1) |
| Cyclical rectal bleeding (n, %) | 11 (13.3) |
